# Fluid responsiveness-guided individualized strategy for preventing hypotension after spinal anesthesia in cesarean delivery: a randomized trial of prehydration versus norepinephrine preinfusion

**DOI:** 10.1186/s12871-026-03672-8

**Published:** 2026-02-12

**Authors:** Chu-Chu Du, Wei Chen, Hao Wang, Ling Guo, Bin Wang, Yu-Long Wang, Yong-Quan Chen

**Affiliations:** Department of Anesthesiology, the First Affiliated Hospital of Wannan Medical College, Wuhu, 241000 China

**Keywords:** Spinal Anesthesia-Induced hypotension (SAIH), Fluid responsiveness, Prophylactic fluid loading, Norepinephrine infusion, Corrected flow time (FTc)

## Abstract

**Objective:**

This study aimed to evaluate the efficacy of fluid responsiveness-guided strategies for preventing spinal anesthesia-induced hypotension (SAIH) in parturients undergoing cesarean delivery, comparing prophylactic fluid loading with norepinephrine infusion.

**Methods:**

In this fluid responsiveness-based stratified randomized controlled trial, eligible parturients were stratified into fluid responsive (FR(+)) and non-fluid responsive (FR(-)) cohorts according to carotid corrected flow time (FTc). Each stratum had a preset sample size of 236, and within each stratum, participants were randomly assigned to receive either prophylactic colloid infusion (Co) or norepinephrine infusion (NE), ultimately forming four subgroups: FR(+)/Co, FR(+)/NE, FR(-)/Co, and FR(-)/NE.Primary outcomes included the incidence of SAIH and maximum reduction in mean arterial pressure (MAP). Secondary outcomes encompassed neonatal umbilical cord blood gas analysis, Apgar scores, intraoperative hemodynamic changes, and postoperative recovery parameters.

**Results:**

Among fluid-responsive parturients, prophylactic fluid loading (FR(+)/Co) and norepinephrine infusion (FR(+)/NE) demonstrated comparable efficacy in preventing SAIH (16.7% vs. 15.1%, *P* > 0.05). In contrast, non-fluid-responsive parturients receiving fluid loading (FR(-)/Co) had a significantly higher SAIH incidence (34.2%) compared to those receiving norepinephrine (FR(-)/NE, 13.6%, *P* = 0.0004). Neonatal umbilical cord blood lactate levels were higher in the FR(+)/NE group compared to the FR(+)/Co group (1.66 ± 0.25 mmol/L vs. 1.55 ± 0.28 mmol/L, *P* = 0.006), suggesting potential fetal hypoxia. Additionally, the FR(+)/NE group exhibited prolonged postoperative gastrointestinal recovery times compared to the FR(+)/Co group (29 [20, 39] h vs. 24 [16, 35] h, *P* = 0.0438).

**Conclusion:**

Fluid loading is optimal for fluid-responsive parturients, whereas norepinephrine infusion is superior for non-fluid-responsive parturients. These findings advocate for fluid responsiveness-guided individualized SAIH prevention strategies to optimize maternal and neonatal outcomes.

**Trial registration:**

This trial was prospectively registered at ClinicalTrials.gov (ChiCTR2400084392) on May 15, 2024, prior to participant enrollment.

## Introduction

Spinal anesthesia, favored for its rapid onset, reliable analgesia, and excellent muscle relaxation, has become the preferred anesthetic technique for cesarean sections. However, this method frequently leads to spinal anesthesia-induced hypotension (SAIH) in parturients, with an incidence ranging from 29% to 80% [1–4]. SAIH manifests clinically as nausea and vomiting, and carries serious implications for both mother and fetus. It can lead to maternal complications such as cerebral hypoperfusion, cardiovascular events, and renal impairment, as well as fetal risks including intrauterine hypoxia and acidosis, thereby jeopardizing maternal and neonatal safety [5–7].

Currently, prophylactic vasopressor administration and fluid loading are the primary clinical strategies for preventing SAIH [7–10]. Among vasopressors, α_1_-adrenergic receptor agonists such as phenylephrine and norepinephrine are recommended as first-line agents. However, their prophylactic use is limited in parturients with preeclampsia, hypertension, or cardiac comorbidities. Although some studies suggest that pre-anesthetic colloid infusion can reduce the incidence of SAIH [11], recent evidence indicates that fluid therapy alone is less effective than vasopressors and insufficient for SAIH prevention [7, 10, 12]. The underlying reasons for this discrepancy remain unclear, and we hypothesize that it may be related to individual variations in fluid responsiveness among parturients.

Fluid responsiveness refers to the capacity of the heart to increase its cardiac output in response to fluid administration, primarily reflecting cardiac preload. It is a well-established concept in the field of fluid resuscitation for critically ill patients [13, 14]. This concept is based on the Frank-Starling mechanism: when a patient operates on the ascending limb of the Frank-Starling curve, increasing preload significantly enhances cardiac output, indicating positive fluid responsiveness [15]. Building on this principle, we propose an innovative hypothesis: fluid loading is effective in preventing SAIH only in parturients with positive fluid responsiveness, and its efficacy is non-inferior to prophylactic norepinephrine administration. This hypothesis may provide a novel approach to individualized SAIH prevention. Corrected flow time (FTc) is a dynamic monitoring technique based on Doppler ultrasound that is non-invasive and can be performed at the bedside. It assesses a patient’s preload status and predicts fluid responsiveness by measuring and heart rate-correcting the carotid artery ejection time. Evidence confirms that changes in cardiac preload and output directly translate to carotid blood flow variations [16–18]. Consequently, carotid ultrasound parameters, particularly the FTc which reflects preload, serve as a reliable predictor of volume responsiveness in spontaneously breathing patients. And monitoring and managing fluid responsiveness can significantly reduce the incidence of hypotension following anesthesia [19].

In this study, we employ the emerging parameter of corrected flow time (FTc) measured by carotid ultrasound to assess fluid responsiveness. By comparing the clinical outcomes of parturients with positive and negative fluid responsiveness who receive either prophylactic fluid loading or norepinephrine administration, we systematically evaluate the incidence of SAIH, the maximum decrease in mean arterial pressure, intraoperative and postoperative complications, and postoperative recovery quality. Additionally, we focus on neonatal outcomes, including umbilical cord blood gas analysis and Apgar scores, to comprehensively assess the impact of different prophylactic strategies on maternal and neonatal outcomes.

## Methods

### Study design and participants

This prospective, randomized, controlled trial was approved by the Ethics Committee of the First Affiliated Hospital of Wannan Medical College and registered at the Chinese Clinical Trial Registry (Registration No.: ChiCTR2400084392). Written informed consent was obtained from all participants or their legal guardians before the study.

The study enrolled parturients who were scheduled to undergo elective cesarean delivery under spinal anesthesia during the period from May 2024 to March 2025. Patients should refrain from eating solid foods 6–8 h before surgery and avoid drinking clear liquids 2 h before surgery. Inclusion criteria included: singleton pregnancy, age 18–48 years, body mass index (BMI) 20–40 kg/m², American Society of Anesthesiologists (ASA) physical status < III, and gestational age ≥ 37 weeks. Exclusion criteria comprised contraindications to the study drugs, comorbidities such as pregnancy-induced hypertension, diabetes, hyperthyroidism, or epilepsy, carotid artery stenosis > 50%, anatomical variations of the carotid artery, or refusal to participate. Additionally, cases were excluded if the surgical duration exceeded 3 h, the sensory block level was below T8 or above T4, or the anesthesia method was altered intraoperatively.

### Carotid artery FTc measurement and fluid responsiveness criteria

**Fig. 1:**
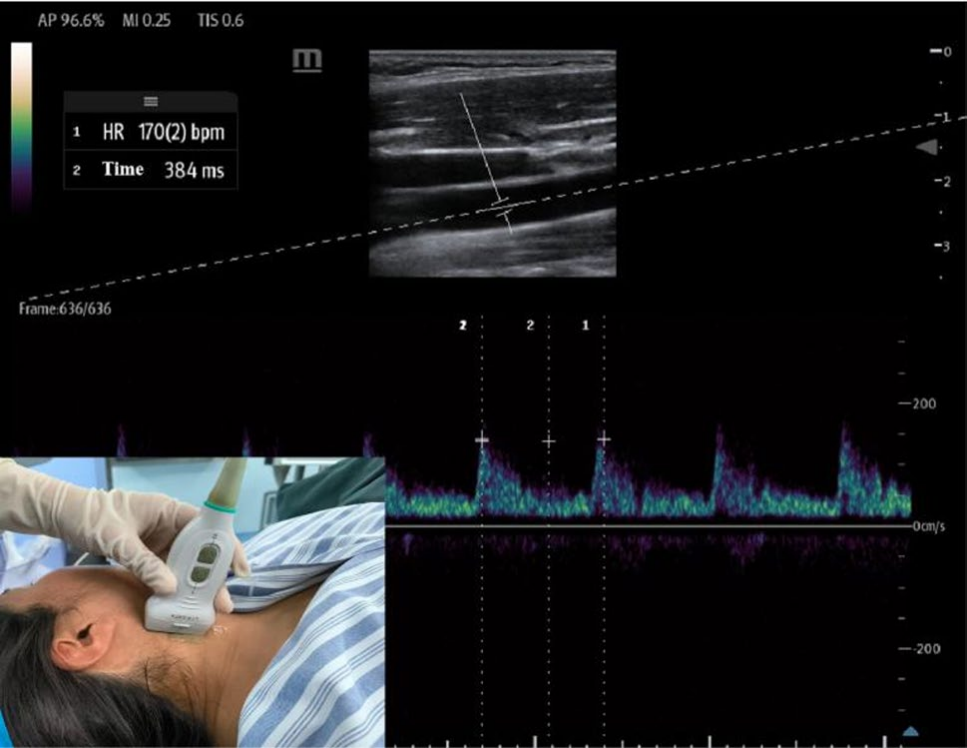
Ultrasound measurement of the blood flow time of the common carotid artery. HR: Heart rate*2; Time: Central blood flow time of the common carotid artery

Fluid responsiveness was assessed by a trained anesthesiologist using FTc (Fig. 1). Briefly, parturients were placed in the supine position with the head rotated approximately 30° to the right to expose the left common carotid artery. A linear array probe (L2-3) was used to perform transverse scanning at the level of the lower edge of the thyroid cartilage. The probe was then rotated 90° to obtain a longitudinal view, and Doppler ultrasound was used to measure the flow time (FT) and heart rate (HR) at the center of the artery, with an insonation angle ≤ 60°. FTc was calculated using the formula: FTc = FT + [1.29 × (HR − 60)]. Based on previous studies [18, 20], fluid responsiveness was defined as FTc < 326.9 ms (positive) or FTc ≥ 326.9 ms (negative).

### Randomization and blinding

This study is a stratified randomized controlled trial based on fluid responsiveness. All enrolled subjects were first stratified according to baseline FTc measurements into fluid-responsive (FR(+)) and non-fluid-responsive (FR(-)) cohorts. The preset sample size for both strata was the same. Subsequently, within each cohort, 1:1 block randomization was performed using a computer-generated random number table: subjects in the FR(+) cohort were randomly assigned 1:1 to either the fluid-responsive colloid group (FR(+)/Co) or the fluid-responsive norepinephrine group (FR(+)/NE); subjects in the FR(-) cohort were similarly randomly assigned 1:1 to either the non-fluid-responsive colloid group (FR(-)/Co) or the non-fluid-responsive norepinephrine group (FR(-)/NE).

**Fig. 2:**
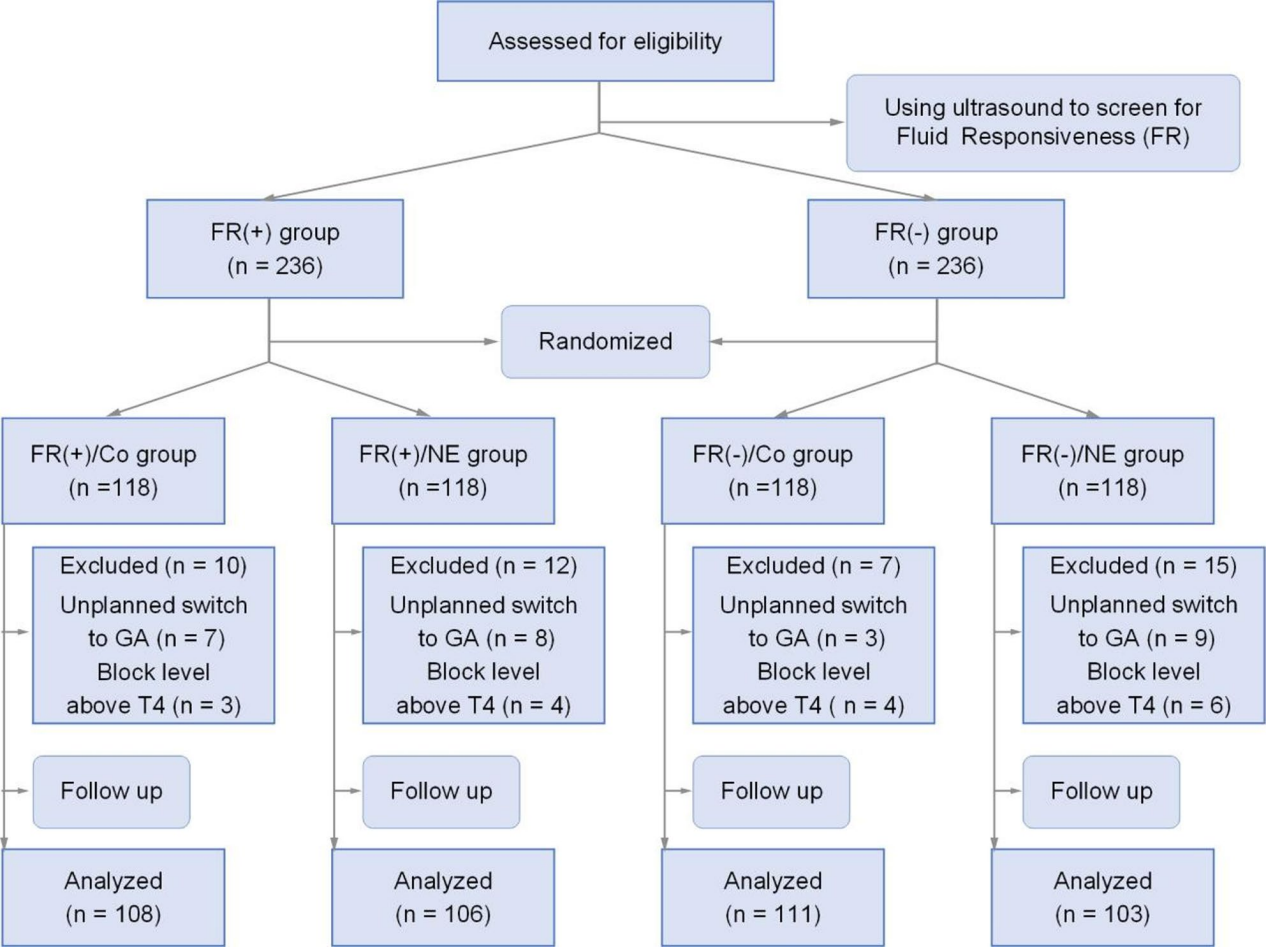
CONSORT flow diagram of participant enrollment, allocation, and analysis. FR(+)/Co: Fluid preload in fluid-responsive parturients; FR(+)/NE: Norepinephrine infusion in fluid-responsive parturients; FR(-)/Co: Fluid preload in non-fluid-responsive parturients; FR(-)/NE: Norepinephrine infusion in non-fluid-responsive parturients

In this prospective study, except for the ultrasound measurers who were involved in subsequent statistical analysis and data processing, the pregnant women, nursing staff, and data collectors were all blinded to the group allocation. The randomization and recruitment process are summarized in the CONSORT flowchart (Fig. 2).

### Interventions and anesthesia protocol

All parturients were transferred to the anesthesia preparation room at least 30 min before surgery, and none had received any sedative medications orally or by injection preoperatively. Standard monitoring was established using an anesthesia monitor, including electrocardiography, non-invasive blood pressure, and pulse oximetry. Baseline blood pressure (BP) and HR were recorded as the average of three consecutive measurements (T0).

#### Colloid group

Before spinal anesthesia, Hydroxyethyl starch 130/0.4 (15 mL/kg) was administered over 30 min [21, 22].

#### Norepinephrine group

Norepinephrine was diluted to 40 µg/mL (2 mg in 50 mL normal saline) and administered as a 0.1 µg/kg bolus followed by a continuous infusion at 0.05 µg/kg/min until delivery, after which the dose was gradually tapered [23].

Spinal anesthesia was performed in the left lateral position at the L3-L4 interspace under ultrasound localization, using 0.75% bupivacaine (7.5–11.25 mg, adjusted for height) combined with 2.5 µg sufentanil, with an injection speed of 1 mL/10 s [24]. After anesthesia, parturients were positioned with a 15° left tilt. Sensory block level was assessed using pinprick testing, and motor block depth was evaluated using the modified Bromage scale [25]. Surgery was initiated when the sensory block level reached T6 or above. The occurrence of hypotension was monitored, and BP and HR were recorded at the following time points: T1 (1 min post-spinal anesthesia), T2 (3 min post-spinal anesthesia), T3 (5 min post-spinal anesthesia), and T4 (at skin incision).

### Outcome measures

#### Primary outcomes

Incidence of SAIH and maximum reduction in MAP. SAIH was defined as a decrease in systolic blood pressure (SBP) of more than 20% from baseline or the presence of hypotension-related symptoms such as nausea and vomiting (which were immediately treated with a 4 µg intravenous bolus of norepinephrine) [26].

#### Secondary outcomes

Neonatal umbilical artery blood gas analysis, 1-minute and 5-minute Apgar scores, intraoperative hemodynamic changes, incidence of intraoperative nausea and vomiting, time to postoperative flatus, and time to ambulation.

#### Safety outcomes

Incidence of intraoperative adverse events and postoperative dizziness or headache.

### Sample size calculation

The sample size was calculated using PASS 15 (NCSS, LLC. Kaysville, Utah, USA) software. Based on previous studies [21, 22], the incidence of SAIH in the prophylactic colloid group was 36.6%. We hypothesized a reduction to 16.6% in the FR(+)/Co group. With a two-sided α = 0.05 and power (1-β) = 0.9, 98 participants per group were required, totaling 392 cases. Accounting for a 20% dropout rate, 472 parturients were enrolled.

### Statistical analysis

Data were analyzed using SPSS 26 (IBM Corporation, Armonk, NY, USA) and GraphPad Prism 9.0 (GraphPad Software, La Jolla, CA, USA) software. Normality was assessed using the Kolmogorov-Smirnov test. Normally distributed data were expressed as mean ± standard deviation (SD) and analyzed using Tukey’s multiple comparisons test; non-normally distributed data were expressed as median (interquartile range [IQR]) and analyzed using Dunn’s multiple comparisons test. These tests were two-tailed, and *P* < 0.05 was considered statistically significant. Categorical data were expressed as frequencies (percentages) and analyzed using chi-square or Fisher’s exact tests. the Bonferroni correction was applied for these multiple comparisons, with an adjusted significance level of *P* < 0.0083.

## Results

### Participant flow and baseline characteristics

A total of 472 parturients meeting the inclusion criteria were screened using carotid artery corrected flow time (FTc) and stratified into fluid-responsive (FR(+) group, *n* = 236) and non-fluid-responsive (FR(-) group, *n* = 236) cohorts. These groups were further randomized into four subgroups: FR(+)/Co group (*n* = 118), FR(+)/NE group (*n* = 118), FR(-)/Co group (*n* = 118), and FR(-)/NE group (*n* = 118). During the study, 27 parturients were excluded due to unplanned changes in anesthesia, and 17 were excluded because the sensory block level exceeded T4. Ultimately, 428 parturients (90.7%) completed the trial, and their data were included in the statistical analysis (Fig. 2).

There were no significant differences among the four groups in baseline characteristics, including age, gravidity, parity, gestational age, BMI, uterine height, abdominal circumference, fetal biparietal diameter, femur length, preoperative fasting time, bupivacaine dosage, time from spinal anesthesia to skin incision, time from skin incision to fetal delivery, or sensory block level (*P* > 0.05, Table 1).

**Table 1:** Demographic and obstetric characteristics

	FR(+)/Co	FR(+)/NE	FR(-)/Co	FR(-)/NE	*P*-value
	(*n* = 108)	(*n* = 106)	(*n* = 111)	(*n* = 103)	
Age (y)	33 ± 5.1	34 ± 4.9	32 ± 4.7	32 ± 4.9	0.158
Gravidity	2 (1, 2)	2 (1.75, 2)	2 (1, 2)	2 (2, 2)	0.612
Parity	1 (0, 1)	1 (0, 1)	1 (0, 1)	1 (0, 1)	0.643
Gestational weeks	38 (37, 39)	38 (37, 39)	38 (38, 39)	38 (38, 39)	0.065
BMI (kg/m^2^)	26.3 ± 3.58	26.6 ± 3.64	26.8 ± 2.99	26.8 ± 3.11	0.619
Fundal height (cm)	34.8 ± 3.33	34.2 ± 2.16	35.0 ± 2.94	34.4 ± 2.38	0.117
Abdominal circumference (cm)	102.0 ± 6.48	102.1 ± 6.72	102.1 ± 6.65	101.1 ± 5.87	0.649
BPD (mm)	89.7 ± 4.74	90.3 ± 4.43	90.9 ± 4.17	90.7 ± 5.29	0.227
FL (mm)	71.0 ± 4.86	71.9 ± 5.83	71.7 ± 4.99	71.8 ± 6.13	0.553
Time from spinal anesthesia to skin incision(min)	13 (10,17)	13 (10,19)	15 (11,18)	13.5 (10,18)	0.739
Time from skin incision to fetal delivery(min)	12 (8,15)	11 (7,14)	11 (9,15)	12 (9,15)	0.143
Fasting time (h)	10.4 ± 2.65	11.1 ± 2.67	10.7 ± 2.70	11.0 ± 2.49	0.227

Data are presented as mean ± standard deviation (SD) or median (interquartile range [IQR]), as appropriate. *Abbreviations* *BMI* Body Mass Index, *BPD* Biparietal Diameter, *FL* Fetal Femur Length

### Primary outcomes

**Fig. 3:**
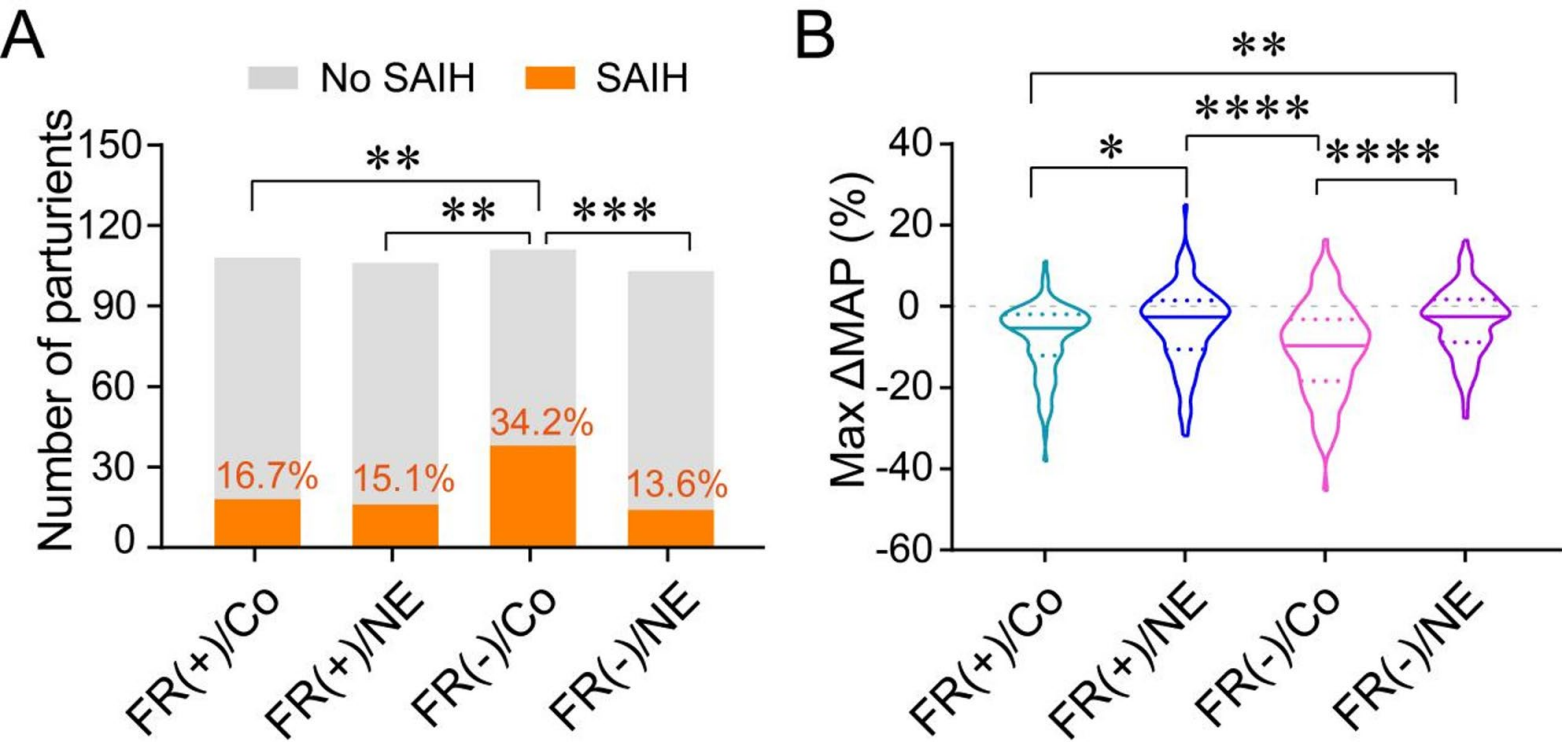
Primary outcomes of the study. **A** Incidence of spinal anesthesia-induced hypotension (SAIH) in the FR(+)/Co, FR(+)/NE, FR(-)/Co, and FR(-)/NE groups. **B** Maximum reduction in mean arterial pressure (Max ΔMAP) expressed as percentage change from baseline: *[(MAPatminimum-MAPatT0)/MAPatT0]×100*. *P < 0.05, **P < 0.01. ***P < 0.001. ****P < 0.0001

The incidence of SAIH in the FR(+)/Co, FR(+)/NE, FR(-)/Co, and FR(-)/NE groups was 16.7%, 15.1%, 34.2%, and 13.6%, respectively. The FR(-)/Co group had a significantly higher incidence of SAIH compared to the other three groups (*P* < 0.0083, Kruskal-Wallis test), while no significant differences were observed among the FR(+)/Co, FR(+)/NE, and FR(-)/NE groups (*P* > 0.05, Fig. 3A).

The maximum reduction in MAP after spinal anesthesia was − 5.4% (-1.9%, -12.1%) in the FR(+)/Co group, -2.5% (1.5%, -10.1%) in the FR(+)/NE group, -9.7% (-3.2%, -18.4%) in the FR(-)/Co group, and − 2.6% (1.7%, -8.9%) in the FR(-)/NE group. The FR(-)/Co group exhibited a significantly greater reduction in MAP compared to the FR(+)/Co and FR(-)/NE groups (*P* < 0.001, Kruskal-Wallis test). Additionally, the FR(+)/Co group showed a greater reduction in MAP compared to the FR(+)/NE and FR(-)/NE groups (*P* < 0.05, Kruskal-Wallis test, Fig. 3B).

### Secondary outcomes

The neonatal umbilical cord blood lactate (Lac) levels were 1.55 ± 0.28 mmol/L in the FR(+)/Co group, 1.66 ± 0.25 mmol/L in the FR(+)/NE group, 1.57 ± 0.30 mmol/L in the FR(-)/Co group, and 1.63 ± 0.31 mmol/L in the FR(-)/NE group. The Lac levels in the FR(+)/Co and FR(-)/Co groups were significantly lower than those in the FR(+)/NE group (*P* < 0.05). However, no significant differences were observed among the four groups in other umbilical cord blood gas parameters, including pH, base excess (BE), bicarbonate (HCO₃⁻), glucose (GLU), or 1-minute and 5-minute Apgar scores (*P* > 0.05, Table 2).

**Table 2:** Neonatal umbilical artery blood gas analysis

	FR(+)/Co	FR(+)/NE	FR(-)/Co	FR(-)/NE	*P*-value
	(*n* = 108)	(*n* = 106)	(*n* = 111)	(*n* = 103)	
PH	7.31 ± 0.04	7.32 ± 0.04	7.31 ± 0.03	7.32 ± 0.03	0.74
Lac (mmol/L)	1.55 ± 0.28^a^	1.66 ± 0.25	1.57 ± 0.30^b^	1.63 ± 0.31	0.019
BE (mmol/L)	-2.4 (-3.14, -1.85)	-2.3 (-3.3, -1.41)	-2.3 (-3, -1.49)	-2.6 (-3.13, -1.78)	0.321
HCO_3_^−^ (mmol/L)	23.54 ± 2.78	23.04 ± 2.93	23.14 ± 2.75	22.837 ± 2.73	0.286
Glu (mmol/L)	3.98 ± 0.49	3.94 ± 0.51	4.10 ± 0.54	4.01 ± 0.52	0.124
Apgar score					
1 min	9 (9, 9)	9 (9, 9)	9 (9, 9)	9 (9, 9)	0.257
5 min	10 (10, 10)	10 (10, 10)	10 (10, 10)	10 (10, 10)	0.519

Data are presented as mean ± standard deviation (SD) or median (interquartile range [IQR]), as appropriate. ^a^*P* < 0.01, FR(+)/Co group vs. FR(+)/NE, ^b^*P* < 0.05, FR(-)/Co vs. FR(+)/NE

**Fig. 4:**
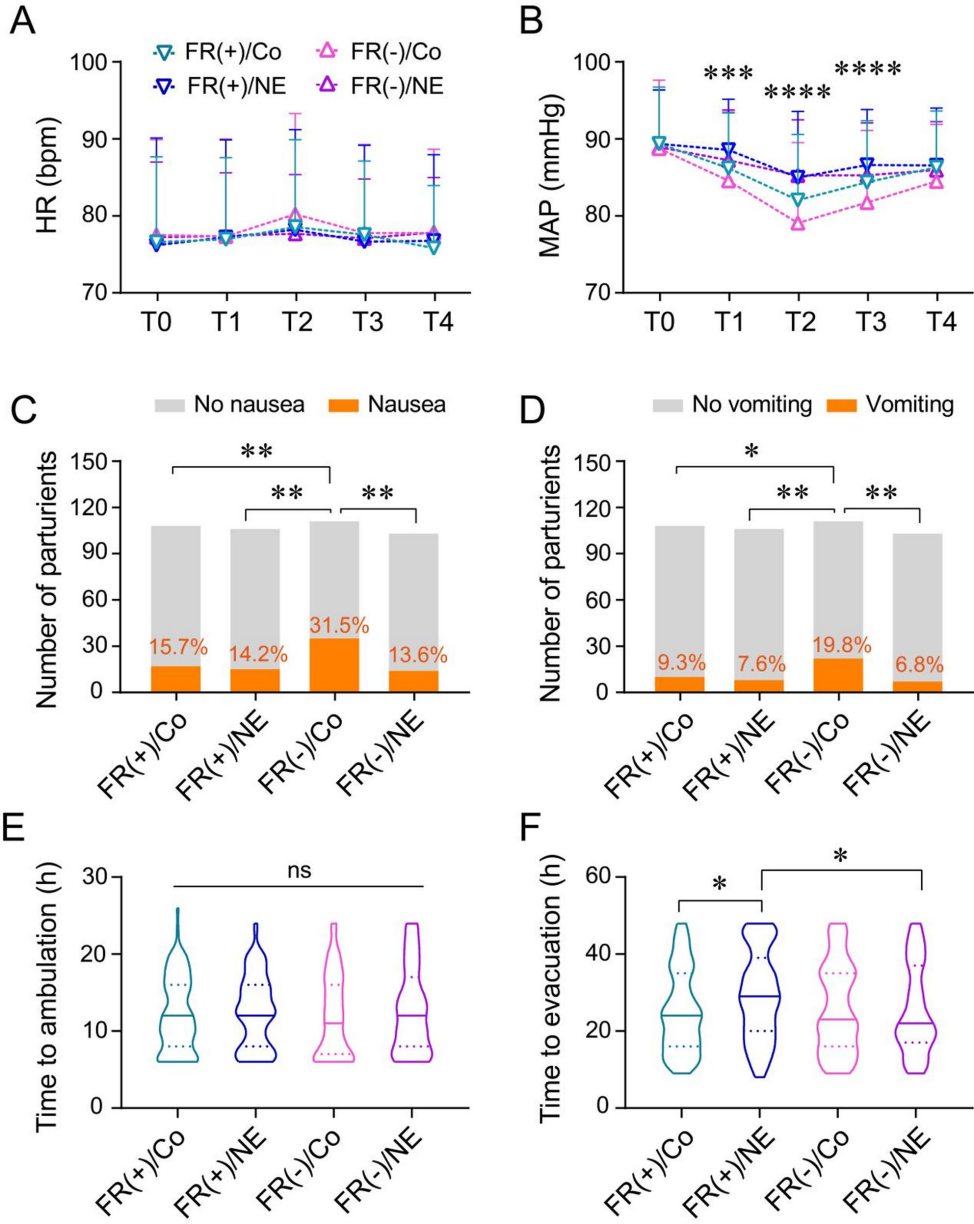
Secondary outcomes of the study. **A** & **B** Heart rate (HR) and mean arterial pressure (MAP) at different time points in the FR(+)/Co, FR(+)/NE, FR(-)/Co, and FR(-)/NE groups. Time points: T0, baseline; T1, 1 min post-spinal anesthesia; T2, 3 min post-spinal anesthesia; T3, 5 min post-spinal anesthesia; T4, skin incision. Data are presented as mean ± standard deviation (SD). ***P < 0.001, ****P < 0.0001 (ANOVA test). **C** & **D** Incidence of nausea (**C**) and vomiting (**D**) in the four groups. Data are presented as number of cases and percentage. *P < 0.05, **P < 0.01 (Chi-square test). Abbreviations: FR(+)/Co, fluid preload in fluid-responsive parturients; FR(+)/NE, norepinephrine infusion in fluid-responsive parturients; FR(-)/Co, fluid preload in non-fluid-responsive parturients; FR(-)/NE, norepinephrine infusion in non-fluid-responsive parturients. **E** & **F** Time to ambulation (**E**) and time to first postoperative evacuation (**F**) in the FR(+)/Co, FR(+)/NE, FR(-)/Co, and FR(-)/NE groups. Data are presented as median (interquartile range [IQR]). ns: *P* > 0.05 (ANOVA test); *P < 0.05 (Dunn’s multiple comparisons test)

Although no significant differences were observed in heart rate (HR) at T0, T1, T2, T3, or T4 among the four groups (*P* > 0.05, Tukey’s multiple comparisons test, Fig. 4A), significant differences in MAP were noted at T1, T2, and T3 (*P* < 0.001, Tukey’s multiple comparisons test, Fig. 4B). The FR(-)/Co group had the lowest MAP, followed by the FR(+)/Co group.

The incidence of intraoperative nausea was 15.7% in the FR(+)/Co group, 14.2% in the FR(+)/NE group, 31.5% in the FR(-)/Co group, and 13.6% in the FR(-)/NE group. The incidence of vomiting was 9.3% in the FR(+)/Co group, 7.6% in the FR(+)/NE group, 19.8% in the FR(-)/Co group, and 6.8% in the FR(-)/NE group. The FR(-)/Co group had a significantly higher incidence of nausea and vomiting compared to the other three groups (*P* < 0.05, Chi-square test, Fig. 4C&D), while no significant differences were observed among the FR(+)/Co, FR(+)/NE, and FR(-)/NE groups (*P* > 0.05, Fig. 4C**&D**).

No significant differences were observed in the time to postoperative ambulation among the four groups (*P* > 0.05, Fig. 4E). However, the time to postoperative evacuation was significantly longer in the FR(+)/NE group compared to the FR(+)/Co group (29 [20, 39] h vs. 24 [16, 35] h, *P* < 0.05, Dunn’s multiple comparisons test, Fig. 4F) and the FR(-)/NE group (29 [20, 39] h vs. 22 [17, 37] h, *P* < 0.05, Dunn’s multiple comparisons test, Fig. 4F).

### Safety outcomes

The incidence of intraoperative adverse events, including hypertension, bradycardia, tachycardia, chest tightness, as well as postoperative dizziness or headache, showed no statistically significant difference (*P* > 0.05, Table 3).

**Table 3:** Other secondary outcomes

	FR(+)/Co	FR(+)/NE	FR(-)/Co	FR(-)/NE	*P*-value
	(*n* = 108)	(*n* = 106)	(*n* = 111)	(*n* = 103)	
Bupivacaine dose (mg)	9 (8.25, 9.75)	9 (8.25, 9.75)	8.25 (8.25, 9)	9 (8.25, 9)	0.114
Block level (T)	6 (6, 7)	6 (6, 7)	6 (6, 6)	6 (6, 7)	0.184
Intraoperative adverse events					
Hypertension	1 (0.9)	0 (0)	0 (0)	2 (1.9)	> 0.05
Bradycardia	6 (5.6)	5 (4.7)	3 (2.7)	5 (4.9)	> 0.05
Tachycardia	6 (5.6)	5 (4.7)	9 (8.1)	6 (5.8)	> 0.05
Chest distress	4 (3.7)	3 (2.8)	7 (6.3)	2 (1.9)	> 0.05
Postoperative adverse events					
Dizziness	6 (5.6)	5 (4.7)	7 (6.3)	6 (5.8)	> 0.05
Headache	0 (0)	0 (0)	1 (0.9)	0 (0)	> 0.05

Data are presented as mean ± standard deviation (SD) or median (interquartile range [IQR]) / n (%), as appropriate

## Discussion

The prevention of SAIH in parturients undergoing cesarean delivery is crucial for maternal and fetal safety. This study demonstrates that in fluid-responsive parturients, prophylactic fluid loading and norepinephrine infusion are equally effective in preventing SAIH. However, in non-fluid-responsive parturients, fluid loading is significantly less effective than prophylactic norepinephrine infusion and is associated with a higher incidence of intraoperative nausea and vomiting. Interestingly, our findings also suggest that prophylactic norepinephrine infusion in fluid-responsive parturients may lead to elevated umbilical Lac levels and prolonged postoperative gastrointestinal recovery time. These results highlight the critical role of fluid responsiveness in determining the efficacy of prophylactic fluid loading for SAIH prevention and its influence on the adverse effects of norepinephrine infusion. Our findings support the implementation of individualized SAIH prevention strategies based on parturients’ fluid responsiveness.

In this study, we utilized the novel ultrasound parameter, FTc. This non-invasive technique offers advantages in terms of simplicity, reproducibility, and clinical feasibility for assessing maternal fluid status. FTc primarily reflects cardiac preload, which is particularly relevant for parturients due to the significant impact of preload on hypotension. Our study confirms that a preoperative FTc < 326.9 ms predicts SAIH with high sensitivity and specificity (> 90%) [20]. Clinically, an FTc increase > 4% indicates positive fluid responsiveness, suggesting a favorable response to fluid therapy [17, 27]. Conversely, minimal changes in FTc suggest negative fluid responsiveness, warranting cautious fluid administration.

This study provides the first evidence of the pivotal role of fluid responsiveness in determining the efficacy of prophylactic fluid loading for SAIH prevention. We observed a significant difference in SAIH incidence between fluid-responsive and non-fluid-responsive parturients receiving prophylactic fluid loading (16.7% vs. 34.2%). The pathophysiology of SAIH involves sympathetic blockade-induced vasodilation, reduced venous return, and exacerbated hypotension due to uterine compression of the inferior vena cava and relative hypovolemia [28]. While vasopressors are commonly used, optimizing effective circulating volume is essential. However, not all parturients benefit from fluid loading [12], as adequate colloid volume is required to prevent hypotension, potentially mediated by fluid responsiveness. In fluid-responsive parturients, increased cardiac output following fluid loading helps maintain stable blood pressure, whereas non-fluid-responsive parturients cannot achieve this benefit, resulting in higher SAIH incidence. Notably, prophylactic fluid loading in fluid-responsive parturients achieved comparable efficacy to norepinephrine infusion. Interestingly, norepinephrine’s preventive effect on SAIH appears independent of fluid responsiveness, likely due to its primary mechanism of increasing peripheral vascular resistance [29, 30].

Consistent with previous studies [23, 26], norepinephrine infusion demonstrated superior SAIH prevention compared to fluid loading when fluid responsiveness was not considered (14.4% vs. 25.6%). Our study revealed higher umbilical cord blood Lac levels in the norepinephrine groups, suggesting potential fetal hypoxia. This effect was more pronounced in fluid-responsive parturients receiving norepinephrine, possibly due to vasoconstriction-induced alterations in fetal hemodynamics and reduced umbilical blood flow, particularly in volume-depleted parturients [31–33]. Nevertheless, the statistical significance of these findings must be interpreted with caution, as their clinical relevance may be limited. Despite these findings, there is insufficient evidence to contraindicate norepinephrine use in fluid-responsive parturients, as no differences were observed in pH, base excess, or Apgar scores. Further investigation is warranted in high-risk populations, such as those with pregnancy-induced hypertension.

Additionally, while no differences were observed in time to ambulation, the FR(+)/NE group exhibited significantly prolonged time to flatus, suggesting that norepinephrine infusion in fluid-responsive parturients may impair postoperative intestinal motility, potentially due to reduced intestinal perfusion in volume-depleted states [34].

It should be noted that the restrictions on the use of hydroxyethyl starch 130/0.4 by agencies such as the European Medicines Agency are mainly based on research evidence suggesting a potential increase in the risk of kidney injury and mortality in critically ill patients, particularly those with sepsis. The subjects of this study were all healthy postpartum women, whose risk-benefit context is significantly different from the aforementioned population. Therefore, the results of this study should be interpreted within this specific context. The findings are consistent with previous clinical studies on pregnant women [35, 36], and did not reveal any significant adverse effects on maternal or neonatal outcomes. Furthermore, a fundamental experiment conducted on rats [37], demonstrated that the drug exhibits a high level of safety at reasonable doses, with a relatively controllable metabolic process. The pharmacokinetic profile of the drug in rats shares similarities with that in humans, providing an important reference for the safety assessment of its clinical application.

This study has several limitations. First, the study did not include a combined prophylactic fluid loading and norepinephrine infusion group. Future studies should explore optimal dosing regimens for combination therapy. Second, we did not assess post-intervention fluid responsiveness, which warrants investigation in subsequent research. Finally, the strict inclusion criteria for elective cesarean delivery may limit the generalizability of our findings to emergency cesarean sections or parturients with pathological conditions such as pregnancy-induced hypertension.

## Conclusions

In conclusion, our study demonstrates that FTc-guided assessment of fluid responsiveness is valuable for optimizing the choice between prophylactic fluid loading and norepinephrine infusion to prevent SAIH, reduce neonatal hypoxia, and promote postoperative recovery. For fluid-responsive parturients, prophylactic fluid loading may be preferable to norepinephrine infusion, while norepinephrine should be prioritized for non-fluid-responsive parturients. These findings provide insights into the variable efficacy of fluid loading in SAIH prevention and contribute to precise perioperative blood pressure management and enhanced recovery after cesarean delivery.

## Data Availability

The data that support the findings of this study are available upon reasonable request from the corresponding author. The data are not publicly available due to privacy or ethical restrictions.

## References

[1] Jaafarpour M, Taghizadeh Z, Shafiei E, Vasigh A, Sayehmiri K. The effect of intrathecal meperidine on maternal and newborn outcomes after Cesarean section: A systematic review and Meta-Analysis study. Anesthesiology Pain Med. 2020;10(2):e100375. 10.5812/aapm.100375 PMC7322789 32637349

[2] McDonnell NJ, Paech MJ, Muchatuta NA, Hillyard S, Nathan EA. A randomised double-blind trial of phenylephrine and metaraminol infusions for prevention of hypotension during spinal and combined spinal-epidural anaesthesia for elective caesarean section. Anaesthesia. 2017;72(5):609–17. 28255987 10.1111/anae.13836

[3] Klöhr S, Roth R, Hofmann T, Rossaint R, Heesen M. Definitions of hypotension after spinal anaesthesia for caesarean section: literature search and application to parturients. Acta Anaesthesiol Scand. 2010;54(8):909–21. 20455872 10.1111/j.1399-6576.2010.02239.x

[4] Yu C, Gu J, Liao Z, Feng S. Prediction of spinal anesthesia-induced hypotension during elective Cesarean section: a systematic review of prospective observational studies. Int J Obstet Anesth. 2021;47:103175. 34034957 10.1016/j.ijoa.2021.103175

[5] Reynolds F, Seed PT. Anaesthesia for caesarean section and neonatal acid-base status: a meta-analysis. Anaesthesia. 2005;60(7):636–53. 15960713 10.1111/j.1365-2044.2005.04223.x

[6] Fantin R, Ortner CM, Klein KU, Putz G, Marhofer D, Jochberger S. [Hypotension induced by spinal anesthesia during Cesarean section: current treatment concepts]. Anaesthesist. 2020;69(4):254–61. 32166396 10.1007/s00101-020-00755-0

[7] Chooi C, Cox JJ, Lumb RS, Middleton P, Chemali M, Emmett RS, Simmons SW, Cyna AM. Techniques for preventing hypotension during spinal anaesthesia for caesarean section. Cochrane Database Syst Rev. 2020;7(7):Cd002251. 32619039 10.1002/14651858.CD002251.pub4 PMC7387232

[8] Kumari K, Chaudhary K, Sethi P, Rathod D, Meshram T, Kothari N, Sharma A, Bhatia P, Singh S. Norepinephrine versus phenylephrine for postspinal hypotension in parturients undergoing Cesarean section: a systematic review and meta-analysis. Minerva Anestesiol. 2022;88(12):1043–56. 35785931 10.23736/S0375-9393.22.16654-X

[9] Baytaş V, Karadağ Erkoç S, Özçelik M, Gökmen D, Bermede AO, Selvi Can Ö, Uysalel A. A Randomized, Double-Blind, graded Dose-Response study of norepinephrine administration for prevention of Post-Spinal hypotension during elective Cesarean delivery. J Clin Med 2023, 12(20). 10.3390/jcm12206437 PMC10607547 37892573

[10] Rijs K, Mercier FJ, Lucas DN, Rossaint R, Klimek M, Heesen M. Fluid loading therapy to prevent spinal hypotension in women undergoing elective caesarean section: network meta-analysis, trial sequential analysis and meta-regression. Eur J Anaesthesiol. 2020;37(12):1126–42. 33109924 10.1097/EJA.0000000000001371 PMC7752245

[11] Ripollés Melchor J, Espinosa Á, Martínez Hurtado E, Casans Francés R, Navarro Pérez R, Abad Gurumeta A, Calvo Vecino JM. Colloids versus crystalloids in the prevention of hypotension induced by spinal anesthesia in elective Cesarean section. A systematic review and meta-analysis. Minerva Anestesiol. 2015;81(9):1019–30. 25501602

[12] Gong RS, Liu XW, Li WX, Zhao J. Effects of colloid preload on the incidence of hypotension in spinal anesthesia for Cesarean section: a systematic review and meta-analysis. Chin Med J. 2021;134(9):1043–51. 33883404 10.1097/CM9.0000000000001477 PMC8116017

[13] Marik PE, Levitov A, Young A, Andrews L. The use of bioreactance and carotid doppler to determine volume responsiveness and blood flow redistribution following passive leg Raising in hemodynamically unstable patients. Chest. 2013;143(2):364–70. 22910834 10.1378/chest.12-1274

[14] Pace R, Lassola S, Miori S, Cammarota G, Barbariol F, Vetrugno L. Carotid vs. aortic velocity time integral and peak velocity to predict fluid responsiveness in mechanically ventilated patients. A comparative study. Minerva Anestesiol. 2022;88(5):352–60. 34761663 10.23736/S0375-9393.21.16035-3

[15] Messina A, Romano SM, Bonicolini E, Colombo D, Cammarota G, Chiostri M, Della Corte F, Navalesi P, Payen D, Romagnoli S. Cardiac cycle efficiency and dicrotic pressure variations: new parameters for fluid therapy: an observational study. Eur J Anaesthesiol 2017, 34(11):755–63. 10.1097/EJA.0000000000000661 28722695

[16] Kim DH, Shin S, Kim N, Choi T, Choi SH, Choi YS. Carotid ultrasound measurements for assessing fluid responsiveness in spontaneously breathing patients: corrected flow time and respirophasic variation in blood flow peak velocity. Br J Anaesth. 2018;121(3):541–9. 30115251 10.1016/j.bja.2017.12.047

[17] Barjaktarevic I, Toppen WE, Hu S, Aquije Montoya E, Ong S, Buhr R, David IJ, Wang T, Rezayat T, Chang SY, et al. Ultrasound assessment of the change in carotid corrected flow time in fluid responsiveness in undifferentiated shock. Crit Care Med. 2018;46(11):e1040–6. 30134304 10.1097/CCM.0000000000003356 PMC6774608

[18] Xu L, Dai S, Shen J, Lv C, Tang Y, Chen X. The predictive ability of carotid artery corrected flow time and respirophasic variation in blood flow peak velocity measured by ultrasonography for fluid responsiveness in parturients for Cesarean delivery. Minerva Anestesiol. 2020;86(10):1039–46. 32538579 10.23736/S0375-9393.20.14315-3

[19] Dai S, Wang C, Tao X, Shen J, Xu L. Predicting fluid responsiveness in spontaneously breathing parturients undergoing caesarean section via carotid artery blood flow and velocity time integral measured by carotid ultrasound: a prospective cohort study. BMC Pregnancy Childbirth. 2024;24(1):60. 38216901 10.1186/s12884-024-06246-z PMC10785346

[20] Kim HJ, Choi YS, Kim SH, Lee W, Kwon JY, Kim DH. Predictability of preoperative carotid artery-corrected flow time for hypotension after spinal anaesthesia in patients undergoing caesarean section: A prospective observational study. Eur J Anaesthesiol. 2021;38(4):394–401. 33122575 10.1097/EJA.0000000000001376

[21] Matsota P, Karakosta A, Pandazi A, Niokou D, Christodoulaki K, Kostopanagiotou G. The effect of 0.5 L 6% hydroxyethyl starch 130/0.42 versus 1 L ringer’s lactate preload on the hemodynamic status of parturients undergoing spinal anesthesia for elective Cesarean delivery using arterial pulse contour analysis. J Anesth. 2015;29(3):352–9. 25266794 10.1007/s00540-014-1926-3

[22] Mercier FJ, Diemunsch P, Ducloy-Bouthors AS, Mignon A, Fischler M, Malinovsky JM, Bolandard F, Aya AG, Raucoules-Aimé M, Chassard D, et al. 6% hydroxyethyl starch (130/0.4) vs ringer’s lactate preloading before spinal anaesthesia for caesarean delivery: the randomized, double-blind, multicentre CAESAR trial. Br J Anaesth. 2014;113(3):459–67. 24970272 10.1093/bja/aeu103

[23] Lyu W, Wei P, Tang W, Ma X, Zheng Q, Zhou H, Zhou J, Li J. Preventing spinal hypotension during Cesarean birth with two initial boluses of norepinephrine in Chinese parturients: A Randomized, Double-Blind, controlled trial. Anesth Analg. 2023;136(1):94–100. 35687059 10.1213/ANE.0000000000006110

[24] Fakherpour A, Ghaem H, Fattahi Z, Zaree S. Maternal and anaesthesia-related risk factors and incidence of spinal anaesthesia-induced hypotension in elective caesarean section: A multinomial logistic regression. Indian J Anaesth. 2018;62(1):36–46. 29416149 10.4103/ija.IJA_416_17 PMC5787888

[25] Lu Y, Zhang Y, Zheng Y, Song Y, Zang Y, Liu Z, Xu Z. Programmed intermittent epidural bolus vs manual epidural bolus for labor analgesia initiation: A randomized Non-Inferiority trial. Drug Des Devel Ther. 2024;18:5063–72. 39529842 10.2147/DDDT.S488920 PMC11552411

[26] Lyu W, Zhang Z, Li C, Wei P, Feng H, Zhou H, Zheng Q, Zhou J, Li J. Intravenous initial bolus during prophylactic norepinephrine infusion to prevent spinal hypotension for Cesarean delivery: A randomized controlled, dose-finding trial. J Clin Anesth. 2024;97:111562. 39047530 10.1016/j.jclinane.2024.111562

[27] Juri T, Suehiro K, Yasuda S, Kimura A, Fujimoto Y, Mori T. Changes in the corrected carotid flow time can predict spinal anesthesia-induced hypotension in patients undergoing Cesarean delivery: an observational study. J Anesth. 2024;38(1):105–13. 38172292 10.1007/s00540-023-03293-2

[28] Humphries A, Mirjalili SA, Tarr GP, Thompson JMD, Stone P. Hemodynamic changes in women with symptoms of supine hypotensive syndrome. Acta Obstet Gynecol Scand. 2020;99(5):631–6. 31856296 10.1111/aogs.13789

[29] Park SK, Park DN, Kim YW, Yoo S, Kim WH, Lim YJ, Park JS, Jun JK, Kim JT. Colloid Coload versus crystalloid Coload to prevent maternal hypotension in women receiving prophylactic phenylephrine infusion during caesarean delivery: a randomised controlled trial. Int J Obstet Anesth. 2022;49:103246. 35012809 10.1016/j.ijoa.2021.103246

[30] Chalkias A. Selective venoconstriction with centhaquine in perioperative and critical care medicine: A Pharmacological lever for enhancing venous return and integrative hemodynamic management. Anesthesiology Perioperative Sci. 2025;3(3):40.

[31] Singh PM, Singh NP, Reschke M, Ngan Kee WD, Palanisamy A, Monks DT. Vasopressor drugs for the prevention and treatment of hypotension during neuraxial anaesthesia for caesarean delivery: a bayesian network meta-analysis of fetal and maternal outcomes. Br J Anaesth. 2020;124(3):e95–107. 31810562 10.1016/j.bja.2019.09.045

[32] Loubert C. Fluid and vasopressor management for Cesarean delivery under spinal anesthesia: continuing professional development. Can J Anaesth = J Canadien D’anesthesie. 2012;59(6):604–19. 10.1007/s12630-012-9705-9 22528166

[33] Mets B. Should Norepinephrine, rather than Phenylephrine, be considered the primary vasopressor in anesthetic practice? Anesth Analg. 2016;122(5):1707–14. 27101504 10.1213/ANE.0000000000001239

[34] Park HS, Choi WJ. Use of vasopressors to manage spinal anesthesia-induced hypotension during Cesarean delivery. Anesth Pain Med. 2024;19(2):85–93. 10.17085/apm.24037 PMC11089295 38725163

[35] Yokoyama N, Nishikawa K, Saito Y, Saito S, Goto F. [Comparison of the effects of colloid and crystalloid solution for volume preloading on maternal hemodynamics and neonatal outcome in spinal anesthesia for Cesarean section]. Masui Japanese J Anesthesiology. 2004;53(9):1019–24. 15500103

[36] Khosravi F, Alishahi M, Khanchemehr Y, Jarineshin H. A comparison between the effects of preloading with ringer’s solution and Voluven on hemodynamic changes in patients undergoing elective Cesarean section under spinal anesthesia. Med Archives (Sarajevo Bosnia Herzegovina). 2019;73(1):44–8. 10.5455/medarh.2019.73.44-48 PMC6445617 31097860

[37] Xu X, Yang H, Chi J, Sun H, Meng X, Gu J. Pharmacokinetics, tissue distribution and metabolism of hydroxyethyl starch 130/0.4 in rats. Int J Biol Macromol. 2025;309(Pt 1):142684. 40169042 10.1016/j.ijbiomac.2025.142684

